# Osteopontin as a Link between Inflammation and Cancer: The Thorax in the Spotlight

**DOI:** 10.3390/cells8080815

**Published:** 2019-08-02

**Authors:** Anne-Sophie Lamort, Ioanna Giopanou, Ioannis Psallidas, Georgios T. Stathopoulos

**Affiliations:** 1 Comprehensive Pneumology Center and Institute for Lung Biology and Disease, University Hospital, Ludwig-Maximilians University of Munich and Helmholtz Center Munich, Member of the German Center for Lung Research, Max-Lebsche-Platz 31, 81377 Munich, Bavaria, Germany; 2 Laboratory for Molecular Respiratory Carcinogenesis, Department of Physiology, Faculty of Medicine, University of Patras, Biomedical Sciences Research Center, 1 Asklepiou Str., University Campus, 26504 Rio, Achaia, Greece; 3 Lungs for Living Research Centre, UCL Respiratory, University College London, London WC1E6BT, UK

**Keywords:** OPN, secreted phosphoprotein 1, SPP1, lung cancer, inflammation

## Abstract

The glycoprotein osteopontin (OPN) possesses multiple functions in health and disease. To this end, osteopontin has beneficial roles in wound healing, bone homeostasis, and extracellular matrix (ECM) function. On the contrary, osteopontin can be deleterious for the human body during disease. Indeed, osteopontin is a cardinal mediator of tumor-associated inflammation and facilitates metastasis. The purpose of this review is to highlight the importance of osteopontin in malignant processes, focusing on lung and pleural tumors as examples.

## 1. Introduction

Osteopontin (OPN), also called secreted phosphoprotein 1 (SPP1), is encoded by the human gene *SPP1* and its murine counterpart *Spp1* [1,2]. Alternative splicing of the human OPN transcript is producing three isoforms called OPN-a, OPN-b, and OPN-c [3] that can be secretory or intracellular [4,5]. OPN is a glycoprotein and presents different post-translational modifications according to the tissue of expression [6,7]. The biological functions of OPN are diverse and specific to a spectrum of physiological and disease conditions. In physiological processes such as wound healing, secreted OPN is involved in cell adhesion to the extracellular matrix via its integrin-binding sequence [8,9], whereas intracellular OPN is involved in mitosis [5]. During chronic inflammation associated with infection and cancer development, OPN regulates host immunity [10] and is a poor prognosticator of survival by enhancing the proliferation and by preventing apoptosis of tumor cells [11]. In non-small cell lung cancers (NSCLCs), OPN induces VEGF expression and facilitates disease progression [12]. Altogether, the multifaceted functions of OPN render it a potential biomarker and therapeutic target, as well as a molecule for which new research tools such as neutralizing antibodies and small interfering RNAs are constantly developed [13,14]. The first part of this review focuses on the OPN gene, the protein isoforms, and their tissue distribution. Subsequently, OPN functions in health and disease are summarized with an emphasis on inflammatory and malignant processes of the chest. Finally, the diverse tools and strategies developed to study OPN biology are discussed.

## 2. Materials and Methods

The knowledge base of this review is based on PubMed searches performed for the present manuscript (https://www.ncbi.nlm.nih.gov/pubmed/; accession date: 14.11.2018). The first search was done using the terms “(osteopontin OR SPP1 OR OPN OR (secreted phosphoprotein 1)) AND cancer” and the limit of publication date within the last five years, yielding 876 results, of which the top 50 sorted by relevance and the top 50 sorted by date were manually reviewed. Their titles and abstracts were studied, and 29 publications were selected for full-text review. Another search employed the terms “(osteopontin OR SPP1 OR OPN OR (secreted phosphoprotein 1)) AND (lung cancer)” and the limit of publication date within the last five years, yielding 128 results, of which the top 50 sorted by relevance and the top 50 sorted by date that was non-redundant with the first search were manually analyzed by screening their titles and abstracts, and 38 papers were selected for full-text review. A final search using the terms “(osteopontin OR SPP1 OR OPN OR (secreted phosphoprotein 1)) AND inflammation” and the limit of publication date within the last five years yielded 514 results, of which the top 50 sorted by relevance and the top 50 sorted by date were manually reviewed. Their titles and abstracts were also screened, and 38 publications were selected for full-text review. Additional searches were performed in the U.S. Clinical Trial Database (https://clinicaltrials.gov/; date of accession: 14.11.2018) using the same terms, providing 25 registered trials, all of which were fully reviewed.

## 3. Review

### 3.1. Physiology of Osteopontin in Humans and Experimental Animals

#### 3.1.1. Osteopontin Genes, Transcripts, and Proteins

**Figure 1:**
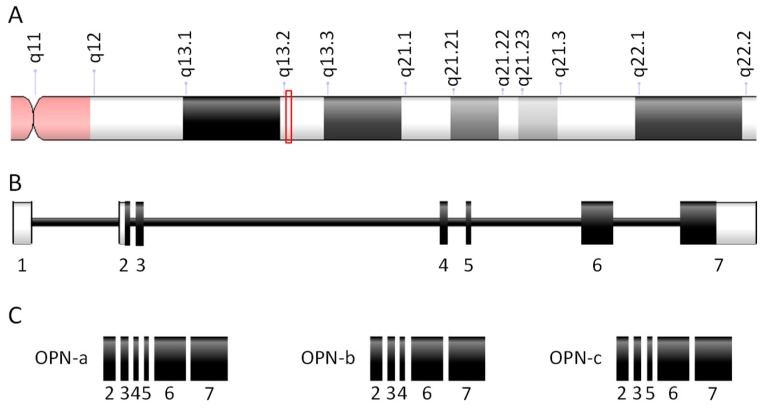
Structure of the human osteopontin (OPN) gene and protein variants. (**A**) Part of chromosome 4 with the *SPP1* locus (red rectangle). (**B**) Structure of the *SPP1* gene. White boxes, untranslated exons; black boxes, translated exons; black line, introns. (**C**) Exon composition of the protein variants resulting from alternative splicing.

OPN was first identified in malignant cells in 1979 [15]. In normal tissues, OPN was described in 1985 as bone sialoprotein 1 only to be renamed to OPN the following year [16,17]. OPN, also called nephropontin and SPP1, is encoded by the human gene *SPP1* [1]. The *SPP1* gene is located on chromosome 4 in locus 4q13.22 (Figure 1) that is encoding proteins of the small integrin-binding ligand N-linked glycoprotein (SIBLING) family [18,19]. The human *SPP1* gene comprises seven exons that are alternatively spliced producing different variants [1,20]. The alternative splicing of *OPN* was described in 2006 with three alternatives forms of the protein [21]. *OPN-a* is considered the canonical transcript, *OPN-b* variant is lacking exon 5, and *OPN-c* variant is missing exon 4; translation of these variants leads to the expression of three different proteins called OPN-a (314 aa), OPN-b (300 aa), and OPN-c (287 aa) [3]. These proteins are negatively charged due to the presence of multiple aspartate residues and their secondary structures are undefined [22]. The murine *Spp1* orthologue is located on chromosome 5 in locus qE5 [2]. The eight exons which compose the gene correspond to a protein composed of 294 amino acids [23].

#### 3.1.2. Tissue-Restricted Patterns of Osteopontin Expression and Processing

**Figure 2:**
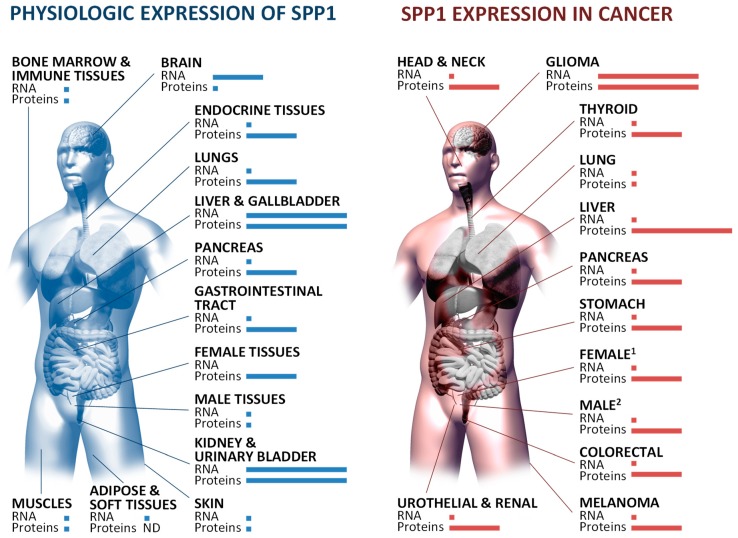
SPP1 expression in physiologic conditions and cancer. *SPP1* mRNA expression was ranked as low, medium, and high. Low, <500 transcripts per million; medium, 500–1000 transcripts per million; high, >1000 transcripts per million. SPP1 protein expression was ranked as low, medium, and high. Low, expression by <25% of individuals tested; medium, expression by 25–50% of individuals tested; high, expression by >50% of individuals tested. RNA-seq and immunohistochemistry data were from The Human Protein Atlas (https://www.proteinatlas.org/).

The OPN protein is produced in a wide range of tissues all over the body in physiologic and disease conditions (Figure 2). However, only a few cell types can express OPN, such as osteoblasts of osseous tissue [24,25,26,27], fibroblasts and activated macrophages of healing wounds [9,28,29], and dendritic, lymphoid, and mononuclear cells of the immune system [30,31,32,33]. Secretory OPN (sOPN) is present in biologic fluids like human serum (average level of 159 ng/mL) [34], bovine milk (average levels 18-138 mg/L) [35], and human urine (average level of 4 µg/mL) [36]. However, intracellular OPN (iOPN) is also expressed [5,37,38] from an alternative downstream transcription initiation site that results in skipping of the first 16 amino acids that function as a membrane localization signal peptide [5,39]. In the lungs, OPN is observed in the sputum (average level of 53 pg/mL) [40] and bronchoalveolar lavage fluid (BALF; average level of 1.1 ng/mL) [41] of healthy individuals, constitutively secreted by airway epithelial cells and alveolar macrophages and exerting diverse functions dependent on its phosphorylation status [42]. In general, sOPN presents different and specific patterns of post-translational modifications according to its cell of origin. For instance, human milk OPN exhibits 36 phosphoserines, 2 phosphothreonines, and 5 O-glycosylated threonines [6], whereas HEK293T cell-derived OPN displays 26 phosphothreonines and 7 O-glycosylated threonines [7]. Unfortunately, there is a lack of information regarding the post-translational modification status of OPN in the lungs. Little information is available and concerns five O-glycosylations, as well as their location and their function [43]. Post-translational OPN modifications result in different functions, but despite the growing literature on the topic, OPN structure–function relationships remain incompletely understood.

#### 3.1.3. Regulation of Osteopontin Expression

OPN expression is regulated by various transcription factors (Table 1) which can link regulatory sequences such as AML-1 and C/EBPα that bind to the E-box motif in the region −170 to −127 of the *SPP1* promoter [44]. Other potential regulatory sequences in the *SPP1* promoter have been identified, including a TATA-like sequence (nucleotides −27 to −22), vitamin-D-responsive (VDR)-like motifs (nucleotides −1892 to −1878 and −698 to −684), an AP-1 binding sequence (TGACACA, nucleotides −78 to −72), and an Ets-1 motif (nucleotides −47 to −39) [45]. Diverse stimuli regulate OPN expression including mechanical stress, hormones, inflammatory mediators, and others listed in Table 1. For instance, in alveolar macrophages, the E3 ligase von Hippel–Lindau protein (VHL) promotes OPN expression by epigenetic modifications [46]. On the contrary, in lung adenocarcinoma-derived A549 cells, the transcription factor Smad4 can bind the OPN gene promoter and inhibit its transcription [47].

In the mouse, similar regulators exist, such as vitamin D3 that can downregulate OPN [58] or transcription factors that can activate OPN expression via promoter binding [59].

#### 3.1.4. Osteopontin as a Matricellular Component

OPN is a significant component of the extracellular matrix (ECM) that regulates matrix interactions and cell adhesion. OPN interacts with ECM elements like collagen and fibronectin, as well as with calcium due to its Ca^2+^ binding sequence [60]. For cell adhesion, OPN possesses integrin-binding sites—an RGD sequence in position 159–161 recognized by integrins αvβ1, αvβ3, αvβ5, α5β1, and α8β1, and the SVVYGLR motif (amino acids 162–168) bound by integrins α9β1, α4β1, and α4β7 [8]. Moreover, the C-terminus of OPN is recognized by the CD44 splice variants CD44v3, CD44v6, and CD44v7 [8]. OPN cleavage by matrix metalloprotease 3 (MMP3), MMP7, and thrombin renders the above motifs more accessible and enhances binding efficiency [4,61]. In lung cancer, the absence of integrin β3 allows OPN-a to stimulate cell growth via the CD44/NFκB pathway, and at high levels of integrin β3 OPN-a inhibits lung cancer cell growth [62].

#### 3.1.5. Osteopontin as a Secreted Protein

OPN secreted by immune cells promotes angiogenesis by binding integrin αvβ3 and activating the PI3K/AKT and ERK pathways on endothelial cells, thereby stimulating VEGF production [63]. Myeloid-derived sOPN also drives tissue remodeling by enhancing cell proliferation and metabolic activation. There are conflicting reports regarding sOPN’s impact on macrophage polarization. Some of them show an M1 polarization mediated by OPN in osteoarthritis [64], whereas other research studies describe how M2 polarization is promoted by OPN fragments. This process is the result of an enhanced MMP production that degrades OPN [65]. Another example of M2 polarization mediated by OPN is in lung adenocarcinoma via the upregulation of PD-L1 to enhance the metastatic process of the cancer [66].

#### 3.1.6. Osteopontin as an Intracellular Molecule

Some cells like macrophages and dendritic cells also produce iOPN observed in the cytoplasm at resting conditions and relocating to the submembranous region where it activates its receptors after stimulation. During mitosis, iOPN is also detected diffusely in the nucleus [5].

#### 3.1.7. Osteopontin in Non-Malignant Diseases

OPN is involved in wound healing resolution by increasing macrophage infiltration, transforming growth factor (TGF) β secretion, and fibroblast proliferation, and by modulating MMP expression [67]. In heart failure, myocardial infarction, and peripheral vascular disease, OPN possesses ambivalent functions. Its anti-inflammatory role limits cardiac fibrosis, vascular calcification, and cardiac tissue remodeling via modulation of collagen deposition and inducible nitric oxide synthase (iNOS) inhibition. On the contrary, the pro-inflammatory effects of OPN on the cardiovascular system include the enhancement of toxic nitric oxide metabolites, fibrosis, calcification, and tissue remodeling via chemoattraction of macrophages, endothelial cells, and smooth muscle cells [68]. In healthy lungs, OPN exhibits protective functions against injury by limiting potential toxicity from the neutrophil extracellular trap (NET) defense mechanism. In more detail, during NETosis, neutrophil DNA traps pathogens, thereby also releasing cytotoxic cationic histones. OPN with its anionic structure can bind these histones and limit their toxic effects [42].

### 3.2. Osteopontin Signaling in Inflammation

#### 3.2.1. Inflammatory Signaling Pathways Initiated by Osteopontin

OPN is a key mediator of inflammatory responses mostly functioning as a chemoattractant for immune cells. The main role of OPN during inflammation is to trigger different leucocytes, induce cytokine secretion, and shape the immune response. At sites of injury, OPN can stimulate migration, accumulation, and persistence of macrophages and regulate their cytokine production by promoting Th1 cell-mediated immunity [69]. The interaction of OPN with α4 and α9 integrins and CD44 can promote either macrophage migration or apoptosis [70]. Wound healing studies have shown that OPN is highly expressed during the acute phase of inflammation and regulates tissue remodeling. Particularly in myocardial infarction, the IL-10-STAT3-galectin-3 axis is essential for OPN-producing reparative macrophage polarization that leads to tissue repair by enhancing fibrosis and clearance of apoptotic cells [71,72].

OPN participates in the pathogenesis of inflammatory and immune-mediated diseases and regulates the host response to infections [10]. For example, OPN promotes IL-12 secretion from dendritic cells (DCs) and their response to hepatitis B virus (HBV) infection, at the same time driving IFN-γ, IL-17A, and IL-10 secretion during *Trypanosoma cruzi* infection [31,73]. OPN also drives sepsis-induced acute lung injury by enhancing neutrophil infiltration via ERK and P38 MAPK activation [74]. Moreover, iOPN is implicated in the differentiation of long-lived natural killer (NK) cells with a memory-like phenotype following homeostatic expansion during host defense against pathogens or cancerous tumor formation. Its absence fails to maintain normal NK cellularity and its stimulation by IL-15 increases cell death [75]. However, OPN limits inflammation, tissue injury, and bacterial load during concurrent pneumococcal infection and allergic airway inflammation [76]. It can be upregulated together with leptin, and both of them may regulate apoptosis, adhesion, migration, and activation of eosinophils, in a PI3K and anti-α4 activation manner [77].

Both iOPN and sOPN control the relative abundance of myeloid and lymphoid cells. iOPN limits myeloid progenitor cell proliferation. E-cadherin induces iOPN which activates the Hippo pathway, thereby upregulating OPN mRNA in myeloid progenitors and eventually limiting their expansion. iOPN also exerts proapoptotic functions in the early stages of granulopoiesis. On the contrary, sOPN enhances lymphoid cell expansion through an anti-apoptotic mechanism [37]. 

OPN binds eosinophil-recruiting chemokines inhibiting their antibacterial activity against *Streptococcus pneumoniae* in parallel with sustained allergic inflammation during asthma [76].

OPN activity can also be annihilated by MMP cleavage in patients with autoimmune diseases, inhibiting OPN-driven cell adhesion and migration, and resulting in a less inflammatory phenotype [78].

#### 3.2.2. Chronic Lung Diseases and Osteopontin

Several inflammatory diseases that are associated with airway remodeling depend on OPN signaling. During asthma, pulmonary fibrotic changes are mediated by interleukin-33 (IL-33)-enhanced amphiregulin production by memory T helper 2 cells. Amphiregulin along with epidermal growth factor receptor (EGFR)-signaling directly reprograms eosinophils to an inflammatory state and stimulates them to produce OPN, which acts as a pro-fibrotic mediator during the disease [79]. Arjomandi et al. also demonstrated that, in asthma, OPN is more fragmentated and can enhance the survival and the recruitment of alveolar macrophages [80]. In chronic obstructive pulmonary disease (COPD), OPN is upregulated and associated with exacerbations and may increase the vulnerability of the host defense to bacterial infections by interfering with the function of antimicrobial proteins [81,82,83]. Furthermore, OPN can be released by senescent pulmonary artery smooth muscle cells and contribute to the pathogenesis of pulmonary hypertension. OPN was recently reported to be positively correlated with plasma levels of IL-2 but not IL-4, which has anti-inflammatory effects in COPD and patients with both ischemic heart disease and increased pulmonary artery plasma pressure [84,85]. Although it is known that statins suppress OPN in cancer cells, their function in COPD was recently studied. Particularly, simvastatin was found to initiate downregulation of adenosine signaling which leads to direct inhibition of IL-13-activated STAT6 and finally repression of OPN expression, therefore preventing disease progression [86]. 

OPN has been also implicated in lung injury of different etiologies. Chronic exposure to carbon nanotubes (CNTs) that have been widely used in the last decades can cause serious injury in the lungs and lead to non-curable pulmonary fibrosis [87]. Lung interstitial fibrosis initiates with an acute inflammatory response, which is characterized by recruitment of inflammatory cells and secretion of pro-inflammatory and pro-fibrotic cytokines, chemokines, and growth factors, such as TNF-α, IL-1β, IL-6, MCP-1, and TGF-β1 [88]. After acute inflammatory and fibrotic responses, the pathologic effects transit to chronic inflammatory fibrosis evident by mild inflammation, increased deposition of ECM proteins, and formation of fibrotic foci [89]. The role of OPN was recently described in a mouse model of CNT exposure induced-fibrosis. It was reported that OPN is highly and persistently induced by CNTs in mouse lungs during both the acute and chronic phases of fibrosis and that OPN promotes lung fibrosis through the activation of TGF-β1 signaling and the promotion of myofibroblast differentiation [90]. TGF-β1 and OPN synergy have been also referred to as a possible target for inhibiting radiation-induced pulmonary fibrosis after lung cancer treatment [91]. Moreover, Fra-2 (a Fos member of the AP-1 transcription factor family) is linked to inflammation and fibrosis in the lungs. Particularly, in LPS-induced lung injury, Fra-2 recruits myeloid cells to the injured tissue in an OPN-depended manner, further exacerbating inflammation [92].

While there is still a need to better understand the pathogenesis of chronic lung scarring in idiopathic pulmonary fibrosis (IPF), studies have shown that OPN exerts severe pro-fibrotic effects [93]. White et al. described a panel of three proteins, namely, surfactant protein (SP)-D, matrix metalloproteinase (MMP)-7, and OPN, that can be used to distinguish IPF from other idiopathic interstitial lung diseases [94]. Additionally, a proteomic analysis of the BALF of patients with IPF has revealed strong expression of OPN along with upregulation of pro-fibrotic CCL24 [95].

### 3.3. Impact of Osteopontin on Cancer Development and Progression

OPN has been repeatedly implicated in the development and progression of several types of cancer. In most cancers, high expression levels of OPN or its isoforms are associated with poor prognosis (Table 2).

#### 3.3.1. Impact of Osteopontin on Thoracic Cancers

Several lines of laboratory research, bioinformatics studies as well as meta-analyses have shown that OPN levels can also be considered as a biomarker for diagnosis or as a predicting tool for patient clinical outcome in several types of cancer, such as lung adenocarcinoma, esophageal carcinoma, prostate cancer, melanoma, and colorectal adenocarcinoma. Its expression levels are considered useful for predicting responses to anti-EGFR therapy in patients with triple-negative breast cancer and as a methylation-based biomarker for precise diagnosis and treatment for thyroid cancer [96,97,98,99,100,101,102].

Interestingly, OPN splice variants seem to differentially participate in cancer progression. For example, in thyroid cancer cells OPN-a overexpression stimulates higher matrix calcification and collagen synthesis when compared to overexpression of OPN-b or OPN-c [103]. In breast cancer, OPN-c is expressed in 75% of patients. At the same time, its expression is rising and is correlated with tumor stage in hepatocellular carcinoma (HCC) [104,105].

#### 3.3.2. Osteopontin in Lung Cancer

**Figure 3:**
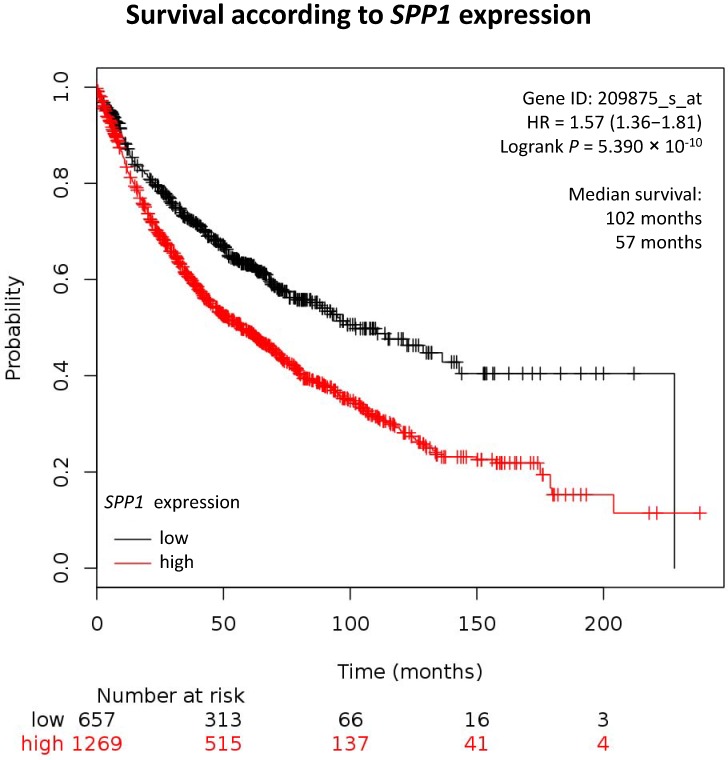
Overall survival in lung cancers according to *SPP1* expression. Data and plot from http://kmplot.com/.

As previously described, in most types of cancer, high levels of OPN are correlated with a more aggressive cancer phenotype and worse prognosis. OPN expression and activity in lung cancer is in accord with this phenotype (Figure 3). In this disease, OPN splice variants have been accused of differential role-playing. In NSCLC, OPN-a promotes a more aggressive phenotype due to the transcription of exon 4 in contrast to OPN-c, where the transcription of exon 4 is absent and is also demonstrated to promote the malignant phenotype of lung tumor cells by enhancing cell-adherent properties to bone tissues [106,107]. OPN-b induces lung cancer cell proliferation, whereas OPN-c enhances their invasive behavior [108]. Investigators have also focused on studying OPN gene single nucleotide polymorphisms (SNPs). These are characterized for their implementation in several types of cancer, but SNPs located in the promoter region of OPN have received investigators’ main focus. While rs11730582 is the most studied SNP of OPN with the −443 CC genotype generally associated with higher expression of OPN and increased cancer risk, this SNP tends to have a better prognosis in NSCLC. On the other hand, the SNP rs17524488 (−156G/G) is associated with higher levels of OPN expression and is correlated to a more advanced stage disease [109,110,111].

In patients with NSCLC, Sun et al. have reported that the combination of OPN and CD44v6 elevated expression of is a valuable independent predictor of tumor recurrence and survival in NSCLC patients [112]. For the same cancer, another study also demonstrated that VEGF and OPN are both overexpressed and positively correlated with tumor progression [12]. In particular, OPN stimulates autocrine and paracrine mechanisms which lead to VEGF accumulation, subsequently promoting tumorigenesis and metastasis [113]. During carcinogenesis, OPN upregulates PD-L1 and M2-polarized tumor-associated macrophages and facilitates tumor immune escape, further enhancing pro-tumor activity [66]. It promotes cancer cell drug resistance and invasion in patients with advanced NSCLC and facilitates the development of small-cell lung cancer (SCLC) cells by inhibiting apoptosis and autophagy [114,115]. Recent studies suggest that fibrocytes that are differentiated from bone marrow-derived CD14^+^ monocytes have features of both macrophages and fibroblasts and may promote cancer progression. They enhance the cancer-stem-cell-like properties of lung cancer cells through the secretion of OPN and its function in the activation of the PIK3K/AKT pathway [116].

Interestingly, lung cancers presenting genomic alterations leading to KRAS activation are characterized by resistance to radiation and poor prognosis. OPN and EGFR act in a synergistic way to initiate stem-like properties of tumor subpopulations whose characteristics are mitosis-like condensed chromatin (MLCC) and high expression of CD133 [117]. In another study, where the transmembrane 4 L6 family member 4 (TM4SF4) known to promote cancer was studied, authors demonstrated that OPN induced by upregulated β-catenin via TM4SF4-driven phosphorylation of glycogen synthase kinase 3b (GSK3β) activated the JAK2/STAT3 or FAK/STAT3 pathway in human lung cancer cells, which also upregulates OPN expression in an autocrine manner [118]. In SCLC patients, OPN has been reported to be one of the most upregulated genes and its inhibition can prevent epithelial–mesenchymal transition (EMT) formation, rendering it a prognostic tool for disease progression [117]. Finally, in patients with lung adenocarcinoma, OPN was identified as a biomarker, and high expression of the protein has been associated with poor patient survival and identified as a predicting tool for disease stage prognosis [96,119].

#### 3.3.3. Malignant Pleural Effusion Promotion by Osteopontin

Malignant pleural effusion (MPE) is a complication of advanced lung, breast, pleural, and other cancers. In most studies performed in the past, enzyme-linked immunosorbent assay (ELISA) and correlations with progression-free survival or overall survival have been used to define OPN expression levels in patients with MPE, emphasizing OPN’s critical role in prognosis [120,121]. Nevertheless, Hsu et al. showed very recently that although pleural fluid OPN along with VEGF and uPA concentrations are elevated in MPE, they are not satisfactory predictors of pleurodesis outcome or patient survival [122]. Moreover, during tumor formation and the interaction of tumor cells with inflammatory responses, mast cells (like several other inflammatory cells) are reported to be recruited in lung adenocarcinomas and interestingly in MPE formation [123]. During these processes, tumor-derived OPN has been reported to promote the pleural fluid formation and pleural tumor progression by inducing vascular hyperpermeability and was recently further described to promote the activation and degranulation of mast cells in the pleural space [123,124]. MPE is a condition that also accompanies malignant pleural mesothelioma (MPM) progression mainly in asbestos-exposed industry workers. Elevated OPN expression levels have been reported during disease progression which is inhibited by zoledronic acid as a single agent in the treatment of MPM [125,126,127,128]. OPN has also been reported to be a target for pemetrexed-based chemotherapy in MPM cells, and its inhibition is accompanied by a reduction in the phosphorylation of AKT [129].

#### 3.3.4. Osteopontin as a Pro-Metastatic Molecule

The mechanisms underlying the aggressive and invasive phenotype of tumor cells associated with OPN expression have been extensively investigated. Although OPN gene silencing has been reported in a study by Sun et al. [130] to contribute to lung tumor cell metastasis by stimulating cell invasion and not cellular migration and proliferation, Polat et al. demonstrated in another study that lung cancer cell proliferation and migration were significantly reduced when OPN was silenced [131]. Moreover, OPN overexpression is shown to activate the PI3K-AKT-Twist pathway, thus promoting EMT and resulting in colorectal cancer metastases [132]. OPN exerts its function by inducing changes in EMT and promoting the initiation of metastases in different types of cancer. OPN can cause an increase in EMT-related transcription factors including Twist, Snail, and Slug [133]. Its overexpression results in serine phosphorylation of Twist, which then binds to the Bmi-1 promoter, subsequently activating EMT in breast cancer cells [134]. Furthermore, OPN can promote EMT-mediated metastases by inducing hypoxia-inducible factor-1 alpha (HIF-1α). Intra-tumor hypoxia stabilizes HIF-1α, which regulates the expression of Twist by binding to the Twist promoter, thus inducing EMT. OPN was shown to increase HIF-1α via the PI3k/AKT pathway in a breast cancer model and induce EMT of HCC cells via increasing vimentin stability, leading to the promotion of HCC metastasis [135,136]. Moreover, a recent study including experiments on liver and lung cancer cells revealed that iOPN translocates to the nucleus and induces mesenchymal-to-epithelial transition (MET) to facilitate the formation of metastases. In particular, nuclear OPN was found to interact with HIF-2α and impact the AKT1/miR-429/ZEB cascade, subsequently enhancing the progression of metastatic colonization. The translocation of OPN into the nucleus is dependent on micro-environmental VEGF activity through a KDR/PLCγ/PKC-dependent pathway [137]. In NSCLC metastasis, OPN plays a key role by inducing cancer-associated fibroblasts (CAFs) [133] and can induce tumor cell migration by interacting with integrins and CD44 [138].

OPN isoforms can differentially facilitate lung metastasis. Tumor-originated iOPN may promote tumor cell survival by preventing tumor-related protein 53 (TP53)-mediated apoptosis, whereas tumor-derived sOPN may function in a paracrine mode to accelerate lung metastasis by enhancing tumor-derived CCL2 chemokine signaling to cognate host receptors [139]. Additionally, MMP-9 activity can lead to an OPN-32 kDa cleaved isoform that induces the production of myeloid-derived suppressor cells, thus mediating tumor immune escape [140]. OPN may also activate ROCK signaling via the FAK/PI3K/AKT pathway, thereby facilitating the invasion of lung cancer cells through lamellipodia formation and the inactivation of cofilin [141].

Finally, lysosomal-associated membrane protein 3 (LAMP3) is a tumor-specific protein induced by hypoxia, which stimulates invasion and metastasis of various cancer cells and has been described to possibly inhibit OPN function by regulating downstream signaling of OPN in osteosarcoma metastasis in the lungs [142]. In another metastatic tumor model, where 4T1 mammary tumor cells are injected into mice, OPN was demonstrated to be required for activating ERK in polymorphonuclear cells in the tumor emboli site and to facilitate tumor colonization through NETosis [143].

### 3.4. Potential Clinical Implications for Osteopontin in Cancer

#### 3.4.1. Osteopontin as a Biomarker

Circulating OPN levels are elevated in several cancers, including mesothelioma [144], colon [145], lung [146], and breast cancer [147]. In malignant mesothelioma (MM), OPN is considered as a potential biomarker and has been quantified in the serum to detect early stages of the disease [148]. More specifically, Pass et al. determined the correlation between MM and increased serum OPN concentrations. They quantified OPN levels in the serum and the tumor tissues in 49 control patients (without any documented exposure to asbestos), 69 asbestos-exposed subjects, and 76 patients with MM [148]. That study showed that patients with or without asbestos exposure had similar OPN levels in the serum. However, in MM, serum OPN concentrations were significantly elevated compared to controls and asbestos-exposed subjects. The serum OPN assay performed by Pass et al. presented a sensitivity of 77.6% and a specificity of 85.5%, denoting OPN as a promising biomarker of MM identification in clinical routine [148]. Other studies revealed conflicting results in the diagnostic efficiency of MM via circulating OPN levels [149,150,151,152,153]. Some of the discrepancies can be interpreted by the origin of the samples used for the OPN assay. The overall diagnostic accuracy of serum OPN to diagnose MM was assessed in a recent systematic review with meta-analysis [150]. That study reported that pooled sensitivity was 57% (95% CI 52–61%), and specificity was 81% (95% CI 79–84%). Moreover, the area under the curve (AUC) of the receiver operating characteristic (ROC) curve was equal to 0.80. Altogether, these results suggest that OPN is a helpful biomarker for MM diagnosis [150].

Contradictory studies exist regarding lung cancer stage and OPN levels. Some studies describe that advanced stages of NSCLC present higher levels of serum OPN compared to the early stages [111]. However, other studies did not detect a significant difference in OPN concentrations according to TNM classification [154,155]. In those studies, the limited number of patients with early-stage disease may be the cause of a lack of statistical significance, which is more compatible with what is observed in daily clinical practice. Other differences could be explained by the tests used for quantification, as well as by thrombin cleavage of OPN during clotting with the consequence of a lower quantifiable OPN amount in serum compared to plasma [154]. Currently, OPN is not an established prognostic or diagnostic biomarker for cancer, and further studies to assess the value of OPN as a biomarker are needed.

#### 3.4.2. Osteopontin as a Therapeutic Target

There are currently no clinical trials targeting OPN as a treatment for lung cancer or mesothelioma (latest review of https://clinicaltrials.gov/ on 1 May 2019). Currently, two studies are recruiting and aim to assess the role of OPN as a biomarker of arteriovenous fistula (NCT03270358) without stenosis and as a risk factor for cerebrovascular stroke (NCT03561285). The only relevant trials are cohort/database studies focused on mesothelioma and aimed at exploring the utility of OPN as a biomarker for mesothelioma.

#### 3.4.3. Tools for Future Research on Osteopontin

In order to study and target OPN, different tools are already available (Table 3). On the one hand, there are tools to produce and functionally analyze OPN truncations. The expression vectors are useful for the overexpression of the protein or the introduction of mutations, while knockout mice models such as the B6.129S6(Cg)-Spp1tm1Blh/J strain [156] are animals without functional OPN. All these tools are allowing the study of OPN influence over organisms or diseases like lung cancer or mesothelioma.

On the other hand, siRNA strategies have also been developed to inhibit OPN. For instance, Zhao et al. showed that a lentivirus-mediated RNAi can reduce the proliferation of the human lung cancer cell line A549 [108]. Blocking antibodies and small molecule inhibitors are also available to neutralize OPN activity. As an example, brefelamide suppresses the in vitro invasion capability of A549 lung cancer cells by inhibiting OPN [14]. The human antibody AOM1 is recognizing the epitope SVVYGLRSKS in human and murine OPN, inhibiting its capability of binding integrins, thereby decreasing the growth of metastatic tumors in a model of non-small lung cancer [13].

## 4. Conclusions and Outlook

Taken together, OPN and its variants are highly involved in the regulation of tumor-associated inflammation as well as the aggressiveness of cancer cells and tumor growth enhancement. Although there are still unknown mechanisms of action of each OPN isoform, it is obvious that OPN variants may be interesting therapeutic targets for chronic inflammatory diseases and cancer. OPN-based clinical tests could be developed to evaluate disease prognosis and response to therapy. Indeed, recent breakthroughs in the targeting of OPN and in the development of tools to analyze the protein are exciting. It is anticipated that soon, new strategies will be developed and significant advancements will be made along these fronts.

## Figures and Tables

**Table 1:** Human OPN regulation. The bold font indicates the regulation process demonstrated in the lung.

Molecule	Activation	Inhibition
Transcription factors	c-Myb [48]	**Smad4** [47]
	ERG [49]	
	AML-1 [44]	
	C/EBPα [44]	
	SP-1 [45] CBFA1 [50] NF-κB [51]	
Hormones	1,25-dihydroxyvitamin D3 [52] Incretins [53]	
Inflammatory mediators	INFγ ^1^ [54]	INFγ ^1^ [52]
	TNFα [54]	INFβ [55]
	IL-6 ^1^ [54]	PGE_2_ [56]
	TGFβ [54]	
	IL-17a [54]	
Other	**Inorganic phosphate** [57] **VHL** [46]	
	Glucose [53]	

^1^ Antagonistic effects depending on cell type and disease.

**Table 2:** Overall survival in different cancers according to *SPP1* expression. Data from http://kmplot.com/. NA indicates not applicable. ↘ and = indicate, respectively, worse and similar survival of patients with high *SPP1* expression compared with patients with low *SPP1* expression. The bold font represents thorax cancers.

Cancer Type	Patient Number	Cut-off	Overall Survival in Months			
			Low *SPP1* Expression	High *SPP1* Expression	Log-rank *P* Value	*SPP1* Effect on Overall Survival
Bladder ^1^	405	929	46	30	5. 80 × 10^−2^	↘
**Breast ^2^**	**3951**	**3485**	**217**	**163**	**1. 00 × 10^−16^**	**↘**
Cervical ^1^	304	788	NA	NA	9. 50 × 10^−4^	↘
Esophageal ^3^	161	1911	45	23	6. 30 × 10^−2^	↘
Gastric	876	2289	31	27	2. 90 × 10^−1^	↘
Head and neck ^1^	500	5017	59	31	1. 00 × 10^−2^	↘
Kidney renal ^1^	530	42,547	118	77	1. 10 × 10^−1^	↘
Kidney renal papillary cell ^3^	288	140,810	87	50	1. 80 × 10^−1^	↘
Liver	364	5242	84	28	3. 50 × 10^−6^	↘
**Lung ^2^**	**1926**	**4151**	**102**	**57**	**5. 30 × 10^−10^**	**↘**
**Lung adenocarcinoma**	**720**	**4305**	**136**	**75**	**1. 30 × 10^−7^**	**↘**
**Lung squamous-cell carcinoma**	**524**	**14,780**	**64**	**33**	**3. 70 × 10^−2^**	**↘**
Ovarian	1435	8960	23	18	7. 70 × 10^−7^	↘
Pancreatic ductal ^4^	177	5866	73	18	5. 90 × 10^−4^	↘
Pheochromocytoma and Paraganglioma	178	223	NA	NA	4. 80 × 10^−1^	=
Rectum ^4^	165	2620	52	37	2. 10 × 10^−2^	↘
Sarcoma	259	516	87	62	5. 40 × 10^−2^	↘
Testicular Germ Cell	134	470	NA	NA	2. 20 × 10^−1^	=
Thymoma	119	208	NA	NA	6. 70 × 10^−2^	↘
Thyroid ^3^	502	308	NA	NA	3. 50 × 10^−1^	=
Uterine ^3^	405	929	104	52	2. 20 × 10^−1^	↘

^1^ squamous-cell carcinoma, ^2^ all histologies, ^3^ carcinoma, ^4^ adenocarcinoma.

**Table 3:** OPN expression vectors. Published plasmids from https://www.addgene.org/.

Organism	Mutation	Isoform	Tag	Name
Human		OPN-a		pDONR223_SPP1_WT_V5 [157]
		OPN-a	GST	pGEX-6P1-OPNa-delta S [21]
		OPN-a		pCR3. 1-OPNa [21]
		OPN-b		pCR3. 1-OPNb [21]
		OPN-b	GST	pGEX-6P1-OPNb-delta S [21]
		OPN-c	GST	pGEX-6P1-OPNc-delta S [21]
		OPN-c		pCR3. 1-OPNc [21]
	Base pairs 49–942	OPN-a	flag	pDest490-OPN-a [158]
	Base pairs 1–175, 217–942	OPN-b	flag	pDest490-OPN-b [158]
	EEKQ-->AAAA	OPN-c		pCR3. 1-OPNc M1 [159]
	EEKQNAV-->AAAAAAA	OPN-c		pCR3. 1-OPNc M3 [159]
	EEKQNA-->EEKNA	OPN-c		pCR3. 1-OPNc M4 [159]
	EEKQNA-->EEKQANA	OPN-c		pCR3. 1-OPNc M5 [159]
	SGSSEEKQNAVSSEET-->AGAAEEKQNAVAAEEA	OPN-c		pCR3. 1-OPNc PSM1 [159]
	SGSSEEKQNAVSSEET-->AGAAEEKQNAVSSEET	OPN-c		pCR3. 1-OPNc PSM2 [159]
	SGSSEEKQNAVSSEET-->SGSSEEKQNAVAAEEA	OPN-c		pCR3. 1-OPNc PSM3 [159]
	SGSSEEKQNAVSSEET-->SGAAEEKQNAVSSEET	OPN-c		pCR3. 1-OPNc PSM4 [159]
	SGSSEEKQNAVSSEET-->SGSSEEKQNAVAAEET	OPN-c		pCR3. 1-OPNc PSM5 [159]
	Base pairs 1–93, 175–942	OPN-c		pDest490-OPN-c [158]
	Base pairs 499–630		flag	pDest490-OPN-10 kDa [158]
	Base pairs 49–498		flag	pDest490-OPN-NT [158]
	Base pairs 631–942		flag	pDest490-OPN-CT [158]
Mouse		opn-1		mOPN-pcDNA [160]
		opn-1		mOPN-PB0 [160]
		opn-2	EGFP	pSpp1-is2 [123]
		opn-3	EGFP	pSpp1-is3 [123]
	antisense			as-mOPN-PB0 [160]
